# A Correction

**Published:** 1911-10-14

**Authors:** Lindsay Johnson


### The Editor's Letter Box.

### A CORRECTION.

*To the Editor of* The Hospital.

Sir,—In your extremely fair criticism of my book "A Pocket Atlas and Text-book of the Fundus Oculi" the reviewer states that the section concerning "606" is no longer quite up to date. The reason is that the book was delayed six months in the press owing to certain difficulties with regard to the coloured plates. I therefore added an appendix on the subject of salvarsan, which was printed and has been inserted in all the later copies issued to the public. The reviewer's copy, being among the first few printed, unfortunately did not contain it.—I remain, yours truly,

Lindsay Johnson.

[The addendum gives a fair and correct description of modern salvarsan treatment, and brings the section in the book thoroughly up to date.—Ed. The Hospital.]

